# Urban Issues: The Sprawl of Food Deserts

**DOI:** 10.1289/ehp.116-a335a

**Published:** 2008-08

**Authors:** M. Nathaniel Mead

The North American urban landscape has changed considerably over the past few decades with the advent of the automobile as the transportation mode of choice. Privatized mobility allowed wealthier people to move outward from city centers toward the suburbs, and with them went many of the supermarkets that used to pervade urban areas. The steady suburbanization of major food retailers is contributing to the emergence of urban “food deserts,” areas within city centers where low-income people have poor access to vegetables, fruits, and other whole foods. Because many chronic diseases have been associated with low consumption of vegetables and fruits, along with high consumption of sugary or high-fat foods, urban food deserts may be taking a health toll on those who live in socially deprived neighborhoods.

Canadian researchers at The University of Western Ontario recently studied the evolution of food deserts since the 1960s in the mid-sized city of London, Ontario. They used a geographic information system (GIS) to map locations of supermarkets in 1961 and 2005. Then they assessed changes in supermarket access in relation to neighborhood location, socioeconomic characteristics, and access to public transit using multiple “network analysis” techniques, which take into account variations in how people are spaced and actually move throughout their environs.

In an article published 18 April 2008 in the online *International Journal of Health Geographics*, the research team reported that low-income residents of London’s inner-city neighborhoods had poorer access to supermarkets than middle- and high-income residents. Moreover, spatial inequalities in access to supermarkets had increased over time. In 1961, more than 75% of London’s inner-city population lived within 1 kilometer of a supermarket, giving them easy access to a variety of foods, says principal investigator Jason Gilliland, who directs the university’s Urban Development Program. In 2005, he says, that number was less than 20%.

“One can say that this problem may only get worse in the near future, considering current concerns about rising food prices and food scarcity,” says Isaac Luginaah, Canada Research Chair in Health Geography at The University of Western Ontario. “[These] findings therefore require policy attention.”

Gilliland suggests several strategies for dealing with urban food deserts. To begin with, he says, cities should support planning policies that boost the inner-city population (e.g., better transportation, housing, and schools) while offering grocery retailers direct incentives (e.g., zoning allowances, tax holidays, or tax rebates) to locate downtown. City planners can also encourage smaller alternative food retailers, especially farmer’s markets. For neighborhoods that cannot support a farmer’s market every day, Gilliland suggests a “mobile market” that visits various neighborhoods throughout the week. For residents without a car, ride sharing and weekend shuttle bus services could be explored to serve disadvantaged neighborhoods without a supermarket.

This is the first known historical analysis of how food deserts evolve over time, exploring empirically (and confirming) the assumption that pedestrians had easier access to grocery stores in the past, says Gilliland. “On the other hand,” he adds, “many people, including policy makers, may assume that accessibility is universal in the age of the automobile, without recognizing the problems faced by people without an automobile.”

Future studies will need to factor in car trips to supermarkets, which the London study did not do, says nutritional epidemiologist Margo Barker of the University of Sheffield School of Medicine and Biomedical Sciences. It remains to be seen, she adds, whether good access to a supermarket actually benefits food decisions and nutritional health, particularly for those most in need.

To improve future studies of these issues, Gilliland says it may be helpful to interview people who live in food deserts to better understand the psychological, economic, and personal effects of these settings. “After all,” he says, “the continued closure of supermarkets in disadvantaged areas will lead to more unemployment and likely have devastating effects on the health of an already vulnerable population.”

## Figures and Tables

**Figure:**
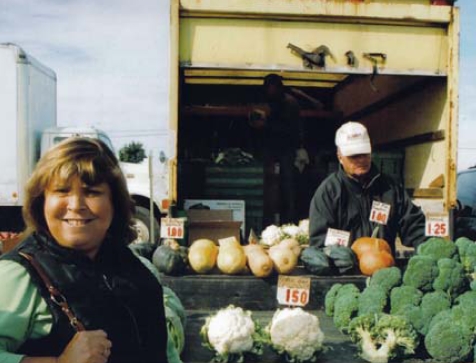
“Mobile markets” can bring fresh produce into urban areas that lack access to well-stocked grocery stores

**Figure:**
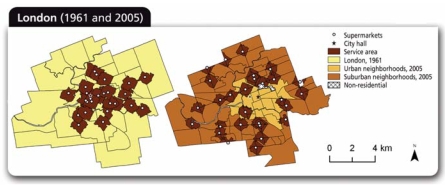
GIS mapping shows the outward migration of supermarkets in a mid-sized Canadian city **Source:** Larsen K, Gilliland J. Int J Health Geogr. 2008 Apr 18;7:16. ©2008 Larsen and Gilliland; licensee BioMed Central Ltd.

